# Predicting the sentiment of South Korean Twitter users toward vaccination after the emergence of COVID-19 Omicron variant using deep learning-based natural language processing

**DOI:** 10.3389/fmed.2022.948917

**Published:** 2022-09-14

**Authors:** Gayeong Eom, Sanghyun Yun, Haewon Byeon

**Affiliations:** ^1^Department of Statistics, Inje University Graduate School, Gimhae, South Korea; ^2^Department of Artificial Intelligence, College of AI Convergence, Inje University, Gimhae, South Korea; ^3^Department of Digital Anti-aging Healthcare (BK21), Graduate School of Inje University, Gimhae, South Korea

**Keywords:** COVID-19 Omicron variant, deep learning, NLP, sentiment analysis, BERT

## Abstract

Although the full vaccination rate of South Korea compared to other countries, concerns about the effectiveness of the vaccine are growing as new COVID variants such as Alpha, Beta, Gamma, Delta, and Omicron appear over time. In this study, we collected Twitter data in South Korea that contained keywords like vaccines after the outbreak of the Omicron variant from 27 November 2021 to 14 February 2022. First, we analyzed the relationship between potential keywords associated with vaccination after the appearance of the Omicron variant in Twitter using network analysis. Second, we developed an efficient model for predicting the emotion of speech regarding vaccination after the COVID-19 Omicron variant pandemic by using deep learning algorithms. We constructed sentiment analysis models regarding vaccination after the COVID-19 Omicron pandemic by using five algorithms [i.e., support vector machine (SVM), recurrent neural networks (RNNs), long short-term memory models (LSTMs), bidirectional encoder representations from transformers (BERT), and Korean BERT (KoBERT)]. The results confirmed that KoBERT showed the best performance (71%) in all predictive performance indicators (accuracy, precision, and F1 score). It is necessary to prepare measures to alleviate the negative factorss of the public about vaccination in the long-term pandemic situation and help the public recognize the efficacy and safety of vaccination by using big data based on the results of this study.

## Introduction

COVID-19 was first reported in Wuhan, China, in 2019, and it is still ongoing worldwide as of May 2022. The World Health Organization (WHO) has declared COVID-19 a “pandemic,” the sixth and highest risk rating, for the third time in history, after Hong Kong flu and swine flu, due to its rapid transmission rate and strong fatality rate (1). As of 22 April 2022, it is counted that 500 million people worldwide have been infected by COVID-19, and 6.2 million have died from it (2). In South Korea, 16 million people (one-third of the total population) were infected, and 21,667 people died from COVID-19 (3). As the COVID-19 Omicron variant has spread in Shanghai, China, after April 2022, the fear of COVID-19 is reviving.

Vaccination may reduce the chance of virus transmission because it can directly protect vaccinated people and inhibit viral shedding (4). In addition, if more than 70% of the members of society are vaccinated, a very effective herd immunity system can be secured (4). Vaccines were developed mainly by global pharmaceutical companies such as Pfizer, Moderna, AstraZeneca, and Janssen (5). Based on these vaccines, South Korea has been rapidly vaccinated: approximately 45 million people (87.7%) received the first dose, approximately 44 million people (86.8%) received the second dose, and approximately 33 million people (64.4%) received the third dose (3). The Korea Disease Control and Prevention Agency (KCCA) reported that as of April 2022, over 80% of the total South Korean population were fully vaccinated (two doses), one of the highest (along with Spain) among 38 OECD member countries (6). Although the full vaccination rate of South Korea is high in the world, concerns about the effectiveness of the vaccine are growing as new COVID variants such as Alpha, Beta, Gamma, Delta, and Omicron appear over time.

The Omicron variant was first discovered in the Republic of South Africa in November 2021, and it was subsequently classified by the WHO as a variant of concern for COVID-19 on 26 November 2021 (7). Furthermore, more than 128 countries have warned of the rapid spread of the Omicron variant. As the Omicron variant has gained dominance rapidly, cluster infections and breakthrough infections are rapidly increasing. Consequently, South Korea is also experiencing the largest outbreak of infection in history. For example, more than 300,000 confirmed cases per day on average are reported in South Korea. The Omicron variant was designated as a dominant variant by the WHO due to its rapid transmission ability compared to the previously prevalent COVID-19 variants. However, a number of studies (8, 9) have reported that the Omicron variant usually had mild symptoms, a low severity rate, and a low mortality rate. As a result, people also have conflicting opinions on vaccination. People have negative or positive opinions. Negative opinions include the “uselessness of vaccination” raised based on the high breakthrough infection rate among confirmed patients (10). Positive opinions argue that vaccination decreases the risk of serious illness and prevents infection.

News media are criticized for lowering credibility and causing confusion due to reckless reporting caused by excessive competition for breaking news and provocative reporting that promotes fear and hatred. Conversely, social network service (SNS) helps users establish social relationships between them through interaction in the internet space. It also influences the opinions and decision-making of the public directly and indirectly. Since users candidly express their opinions on social media (11), it is a useful source for retrieving information on public opinion (12). Consequently, it can be meaningful to understand the social atmosphere related to vaccination. In other words, as opposed to the media, it has been requested to conduct studies by using sentiment analysis (13), which analyzes people’s emotions, attitudes, evaluations, opinions, etc. through SNS posts that have not been filtered in various ways.

In this study, we collected Twitter data in South Korea that mentioned vaccines after the outbreak of the Omicron variant from 27 November 2021 to 14 February 2022. First, we analyzed the relationship between potential keywords associated with vaccination after the appearance of the Omicron variant in Twitter using network analysis. Second, we developed the best model for predicting the emotion of speech regarding vaccination after the COVID-19 Omicron variant pandemic by using deep learning algorithms, such as support vector machine (SVM), recurrent neural networks (RNNs), long short-term memory models (LSTMs), bidirectional encoder representations from transformers (BERT), and Korean BERT (KoBERT).

## Materials and methods

### Data collection and analysis period

**FIGURE 1:**
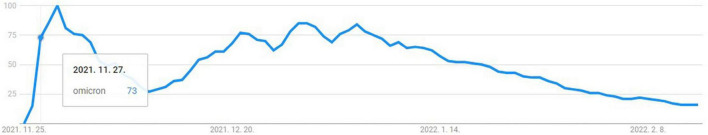
Trend in the volume of keyword search related to the Omicron variant in South Korean news media (27 November 2021—14 February 2022).

The data collection period was determined using Google Trends. In Google Trends, when the number of searches is closer to 100, it is interpreted as higher public interest. The data collection period was set from 27 November 2021 (when the volume of searching the Omicron variant began to increase rapidly) to 14 February 2022 because the COVID-19 Omicron variation continued to spread (Figure 1). The data necessary for this study were collected using snscrape, a Python library. For this study, we collected posts containing “Omicron” and “vaccine,” keywords, on Twitter during the collection period and selected 6,561 posts for analysis through data preprocessing.

### Data preprocessing

**FIGURE 2:**
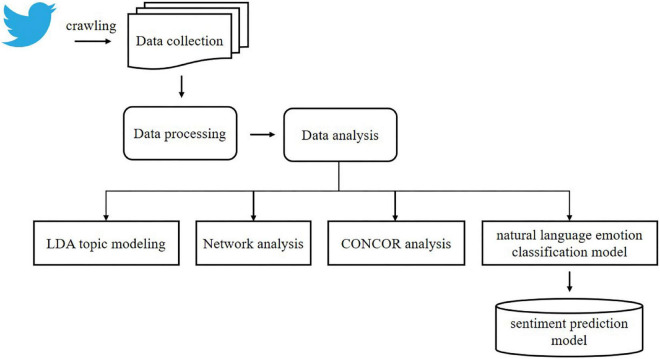
Schematic diagram of this study.

Numbers, alphabetical characters, and special characters were removed from the collected data using KoNLPy, a Python library to process Korean natural language. Likewise, we study extracted noun morphemes through Korean morpheme analysis using okt.nouns in the KoNLPy module and removed posts on topics that did not agree with the objective of this study. We study treated words that appeared frequently but were not necessary for the study as stopwords (e.g., people and country). Then, we judged slang and newly coined words (e.g., lol) as expressions of emotions, so they were not excluded in the data cleanup process and included in the data for analysis. Lastly, we recruited experimental groups (each group was composed of five subjects) and labeled the emotion felt from the collected data as negative (–1), neutral (0), or positive (1). The final data were generated by (1) merging the five datasets after labeling and (2) designating the label found most frequently for each sentence as the final emotion. The schematic diagram of this study is presented in Figure 2.

### Term frequency–inverse document frequency

The term frequency–inverse document frequency (TF-IDF) weighting model is a way to evaluate the importance of a word in a document for text mining. A word with a higher TF-IDF weight is more likely to determine the subject or meaning of the document it belongs to, and this can be used as a yardstick to extract keywords (14).

### LDA topic modeling

Topic modeling is one of the text mining techniques that stochastically extract topics from documents or texts in documents (15). Topic modeling is an analysis technique that extracts topics based on words and automatically extracts specific issues or topics representing the corresponding texts (1). It is useful for analyzing potential topics or issues with news big data (1). The number of topics was determined using the coherence model of the gensim module. The number of topics with the highest coherence score is the appropriate number of topics for topic modeling. We calculated coherence scores, and it was confirmed that four topics were the appropriate number of topics.

### Semantic network analysis

Network analysis is also called semantic network analysis. It analyzes by constructing a keyword network to understand the structure of the concepts and symbols in sentences (16). It treats and utilizes a word frequently used in texts as an indicator revealing the nature of discourse (16). We performed network analysis using Ucinet (17) and identified the connection structure between major keywords.

### CONCOR

CONCOR analysis is an analysis method for deriving relationship patterns between nodes and is used for structural analysis of relationships in subgroups existing in the network (18). This analysis has the advantage of easily identifying the meaning of each group and the structure of the entire network because it clusters and schematizes the entire semantic network centering on the connectivity between words. We conducted CONCOR analysis using Ucinet (17) and NetDraw (17).

### Construction of natural language emotion classification model

We constructed sentiment analysis models regarding vaccination after the COVID-19 Omicron pandemic by using five algorithms (i.e., SVM, RNN, LSTM, BERT, and KoBERT). The order of these algorithms refers to the temporal order of development. This study could analyze and interpret factors, which improved the performance of the high-dimensional computational models that overcome the shortcomings of the previous general models and those of the increased model evaluation scores by developing NLP models using five algorithms because classification models had different aspects in processing natural language. Lastly, we selected the model with the highest performance after comparing and analyzing the predictive performance (e.g., accuracy) of the developed five NLP models. Then, we designed models that could predict emotions by converting negative, neutral, and positive emotions into percentages when an arbitrary sentence was entered.

#### Support vector machine

The SVM algorithm is a non-stochastic binary linear classification model that determines the category in the new data will fall into based on the given dataset, which belongs to either of the two categories, and defines a decision boundary, which is a baseline for classification between categories (19). This model is designed based on a non-stochastic binary classification model. Due to this characteristic, a binary classification model was designed after completing auto-labeling neutral label sentences into positive or negative based on the KNU sentiment lexicon (20). We adopted PolyKernel, the model with the highest accuracy, by using GaussianKernel, PolyKernel, and LinearKernel, built-in functions of the scikit-learn library.

#### Recurrent neural network

**FIGURE 3:**
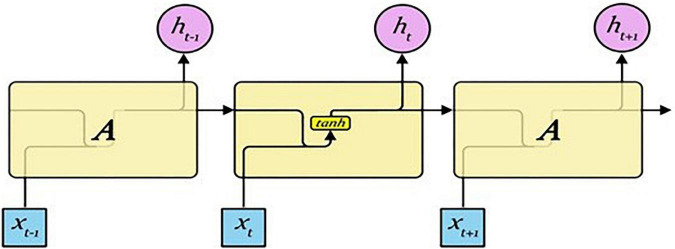
RNN structure.

A RNN is a type of artificial neural network, and it is specialized in processing ordered information like natural language (21). An RNN can process input in the form of a sequence using its internal memory, unlike a forward neural network, because it has a cyclic structure. Therefore, it is a suitable structure for solving natural language processing problems because it can process sequence-type input using internal memory. The structure of an RNN is presented in Figure 3 (22).

#### Long short-term memory

**FIGURE 4:**
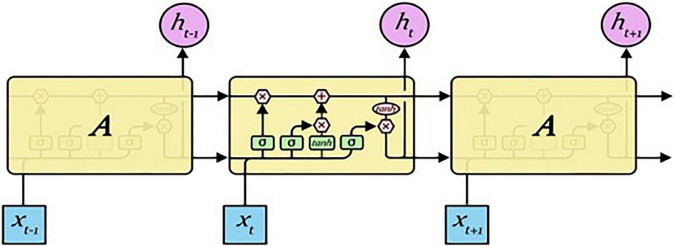
LSTM structure.

LSTM is a model that improves gradient vanishing, gradient exploding, and long-term dependency problems of RNNs. Although LSTM has the characteristics of an RNN, it is different in that it can adjust values by attaching cells, called gates, to the input, forget, and output parts of the RNN. A forget gate decides whether to save previous-state information, an input gate decides whether to save new input information, and an output gate controls the output of an updated cell. The structure of LSTM is presented in Figure 4 (22).

#### Bidirectional encoder representations from transformers

**FIGURE 5:**
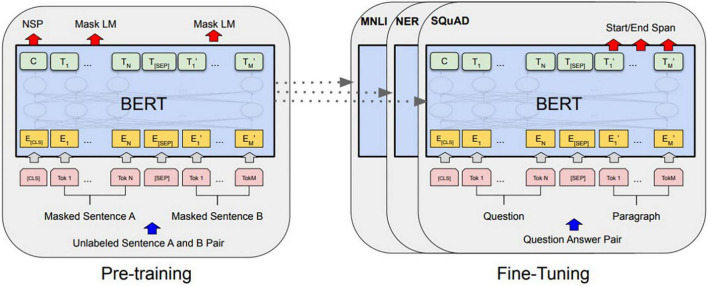
BERT pre-training and fine-tuning procedures.

BERT, developed by Google, is a pre-trained language model, and it performs well in various natural language processing fields, such as question and answer and sentence classification. ELMo (23) and GPT (24) are pre-trained language models similar to BERT, and they predict an (N + 1)th word using the preceding N words (25). Although learning was conducted in one direction, BERT uses the encoder part of the transformer, which can use the contexts in both directions (25). It is the difference between them. The learning process of conventional deep learning models and that of BERT are different in that a BERT model pre-trained on a large amount of corpus can be fine-tuned and applied for other tasks. In other words, since a complex specific task with a large amount of computation is processed at the processing stage of BERT, high prediction performance equivalent to a CNN and LSTM can be achieved even in deep learning models such as DNN and RNN, even if the model is not linked to a high-computational model such as CNN or LSTM. Both tokenization and modeling were used in the built-in functions of the pre-trained Transformer-BERT linkage library (tokenizer = BertTokenizer.from_pretrained, model = TFBertModel.from_pretrained). Figure 5 shows schematic diagrams of the BERT overall pre-training and fine-tuning procedures.

#### Korean bidirectional encoder representations from transformers model

KoBERT was developed to overcome the limitations in analyzing Korean language of the existing BERT. It learned a large corpus consisting of millions of Korean sentences collected from Wikipedia and news. It improved performance by more than 2.6% using only 27% of the previous amount by applying the data-based tokenization technique to reflect the characteristics of irregular changes in Korean (26). Although the description of KoBERT is different from that of BERT, the structure of KoBERT is not different from that of BERT because it is an improved model that overcomes the decreased accuracy of Korean sentence-based model and Korean incompatibility of the bert-base-multilingual-cased Tokenizer, the tokenizer of BERT.

## Results

### Frequency of top keyword and term frequency–inverse document frequency analysis

Table 1 shows the top 30 keywords extracted based on the frequency of keywords in the speech data and the top 30 keywords of TF-IDF. The results of frequency analysis confirmed that “Omicron,” “vaccine,” “inoculation,” “COVID-19,” “infection,” “effect,” “vaccine booster,” “spread,” “proof of vaccination,” and “disease control” were derived in the descending order of magnitude. Although it is possible to infer trends and flows based on the top 30 keywords extracted based on frequency, there is a limit to understanding the importance of each keyword solely based on the results of frequency analysis. We analyzed the importance of keywords more accurately by utilizing TF-IDF, which is a technique of assigning importance to each keyword as a weight to supplement this. TF-IDF analysis found inoculation, COVID-19, infection, effect, vaccine booster, proof of vaccination, spread, disease control, confirmed cases, and Pfizer, in the order of magnitude.

**TABLE 1:** Results of frequency analysis of top 30 keywords and TF-IDF.

	Keywords	Frequency	Keywords	TF-IDF
1	Omicron	8,279	Inoculation	3286.131
2	Vaccine	7,997	COVID-19	2777.284
3	Inoculation	3,246	Infection	2610.349
4	COVID-19	1,860	Effect	2197.277
5	Infection	1,508	Vaccine booster	1998.605
6	Effect	1,129	Proof of vaccination	1890.749
7	Vaccine booster	965	Spread	1781.616
8	Spread	825	Disease control	1757.968
9	Proof of vaccination	820	Confirmed case	1670.345
10	Disease control	719	Pfizer	1640.591
11	Pfizer	703	Research	1463.721
12	Confirmed case	691	Delta	1361.974
13	Research	573	Prevention	1284.294
14	Delta	507	Cold	1253.636
15	Prevention	464	Symptom	1151.075
16	Cold	421	Immunity	1126.171
17	Symptom	385	Unvaccinated people	1104.679
18	Unvaccinated people	378	Mask	1093.829
19	Immunity	367	Government	1077.68
20	Government	363	Treatment	1045.194
21	Mask	361	Antibody	1030.577
22	Treatment	331	Confirmed case	989.9985
23	Antibody	320	Result	989.3775
24	Result	320	Severe symptom	951.5444
25	Confirmed case	316	Examination	911.4365
26	Severe symptom	301	Response	866.7388
27	Examination	267	Breakthrough infection	859.0066
28	Response	261	Death	837.5117
29	Breakthrough infection	259	Completion	827.4268
30	Death	247	Occurrence	822.6

### LDA topic modeling

Table 2 shows the results of LDA topic modeling. Topic 1 (Omicron) consisted of words expressing the symptoms of the Omicron variant such as influenza and cold, and keywords associated with the disease control system such as the proof of vaccination, disease control, and quarantine, and negative keywords such as fear and fatality. Topic 2 was a “vaccine.” It was composed of words related to the COVID-19 vaccine such as “effect,” “inoculation,” “Pfizer,” “vaccine booster,” “COVID-19,” “prevention,” and “antibody,” and words related to virus variant such as “research,” “variant,” and “Delta.” Topic 3 (“vaccine inequality”) consisted of “worldwide,” “spread,” “inequality,” “helplessness,” and “concern,” which implied that there was a problem of unequal delay in vaccination among countries. The keywords of Topic 4 (“breakthrough infection”) were vaccinated people, inoculation, infection, confirmed cases, breakthrough infection, and vaccine, which indicated that it was composed of words implying “breakthrough infection,” which means those who completed vaccination were infected.

**TABLE 2:** Results of LDA topic modeling.

Topic	Topic name	Keywords
1	Omicron	COVID-19, proof of vaccination, influenza, cold, government, disease control, fear, fatality, entry, and quarantine
2	Vaccine	Effect, inoculation, Pfizer, vaccine booster, COVID-19, prevention, antibody, research, variant, and Delta
3	Vaccine Inequality	Research, worldwide, symptom, treatment, spread, COVID-19, inequality, helplessness, concern, and response
4	Breakthrough Infection	Vaccinated people, infection, confirmed case, spread, disease control, COVID-19, definite diagnosis, unvaccinated people, breakthrough infection, and outbreak

### Centrality and network visualization of keywords

**FIGURE 6:**
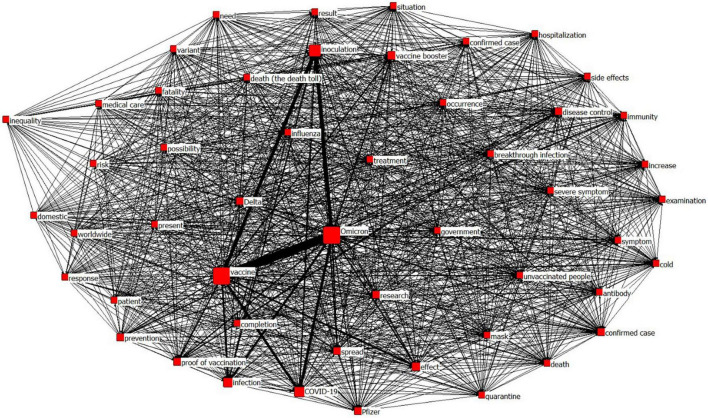
Visualization of network analysis results.

Figure 5 shows the visualization results of keywords using network analysis. Since the size of a node expresses the value of degree centrality, it was confirmed that larger sizes of Omicron, vaccine, and inoculation increased degree centrality. The thickness of the line indicates the frequency of co-occurrence. A thicker line means a higher co-occurrence frequency of two words. Figure 6 confirms that the co-occurrence frequency of Omicron and vaccine, that of vaccine and inoculation, that of inoculation and Omicron, and that of effect and vaccine were high.

### Results of CONCOR analysis

**FIGURE 7:**
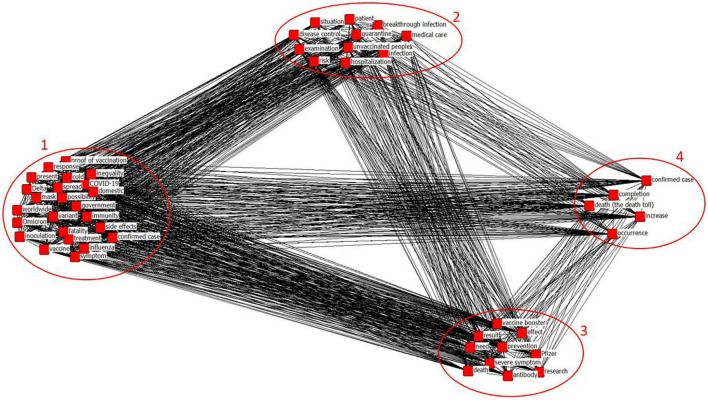
Visualization of CONCOR analysis results.

CONCOR analysis derived four appropriate similarity clusters (Figure 7). The clusters were named based on the derived keywords: Omicron and vaccination status (cluster 1), infection and treatment (cluster 2), vaccine effectiveness and the need for vaccine research (cluster 3), and increase in confirmed cases and deaths (cluster 4). In cluster 1, keywords associated with virus variants such as Omicron, variant, fatality, cold, influenza, and Delta, and those associated with vaccine such as the proof of vaccination, immunity, vaccine, inequality, and inoculation were derived. Cluster 2 showed keywords related to infection statuses such as infection and breakthrough infection, and those related to treatment statuses such as medical care, quarantine, hospitalization, and disease control. Cluster 3 can be inferred as the need for vaccine research on the current virus variant issues from keywords for vaccine effects such as severe symptom, death, prevention, and effect, and keywords such as vaccine booster, Pfizer, research, and necessity. Cluster 4 includes keywords for the current situation in which the number of deaths and confirmed cases was increasing. Table 3 presents keyword factor types based on the CONCOR analysis.

**TABLE 3:** Keyword factor types related to Omicron and vaccine based on CONCOR analysis.

	Cluster name	Keywords
1	Omicron and vaccination status	Omicron, proof of vaccination, COVID-19, variant, immunity, treatment, definite diagnosis, vaccine, possibility, fatality, inequality, government, symptom, spread, cold, worldwide, present, inoculation, response, domestic, mask, Delta, side effects, and influenza
2	Infection and treatment	Medical care, quarantine, hospitalization, situation, examination, breakthrough infection, infection, risk, patient, disease control, and unvaccinated people
3	Vaccine effectiveness and the need for vaccine research	Prevention, need, consequence, death, effect, Pfizer, research, antibody, vaccine booster, and severe symptom
4	Increase in confirmed cases and deaths	Increase, occurrence, death (the death toll), confirmed case, and completion

### Natural language sentiment classification and the results of predictive models

**FIGURE 8:**
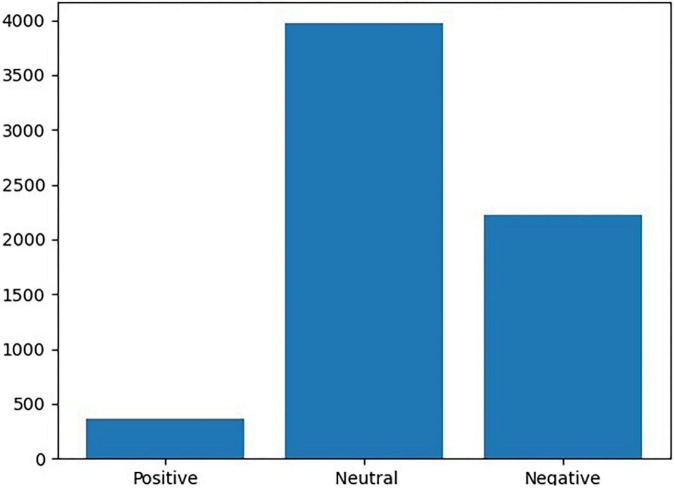
Frequency of positive labels and negative labels.

We set the learning environment to 100 epochs with a batch size of 32 and 20% Test_dataset. Figure 8 shows the label distribution of the data. The results of label distribution analysis confirmed that there were more negative labels than positive labels (363 positive cases, 2,228 negative cases, and 3,970 neural cases).

Table 4 presents the performance (accuracy, precision, and F1 score) of the five sentiment classification models (KoBERT, BERT, LSTM, RNN, and SVM). KoBERT showed the best performance in accuracy, precision, and F1 score, and SVM had the lowest performance. All measures (i.e., accuracy, precision, and F1 score) of the developed predictive models improved when the algorithm of a model was advanced (e.g., from SVM to KoBERT). KoBERT (highest performance) showed 13.864% higher accuracy, 14.704% higher precision, and 15.84% higher F1 score than the SVM (lowest performance). Unlike other models, the RNN and LSTM did not show a difference in predictive performance. Finally, we designed a model for predicting the emotion of a new sentence using KoBERT, which showed the highest accuracy among these sentiment classification models.

**TABLE 4:** Performance evaluation results of five sentiment classification models.

	SVM	RNN	LSTM	BERT	KoBERT
Accuracy	57.677	62.241	62.897	67.182	71.541
Precision	56.312	61.027	60.998	66.445	71.016
F1-score	54.991	59.971	60.212	65.612	70.831

**FIGURE 9:**
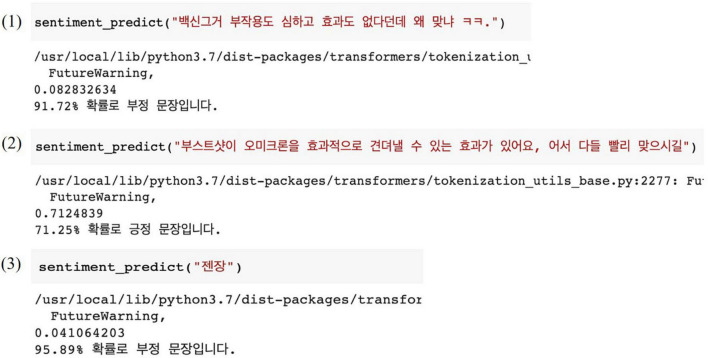
Sentiment prediction result for new speech input of the final sentiment prediction model (KoBERT).

We evaluated the performance of the KoBERT-based sentiment prediction model by entering new speech sentences. Figure 9 shows these results. Speech sentence 1 (“Why do you get vaccinated, yet it is ineffective and has severe side effects?”) was predicted as a negative sentence at a 91.72% probability. It was believed that a new expression such as lol contained negative emotions. Speech sentence 2 (“A vaccine booster helps people withstand Omicron effectively. I hope everyone gets it soon.”) was predicted as a positive sentence, with a 71.25% probability. Speech sentence 3 including slang was predicted as a negative sentence, with a 95.89% probability.

## Discussion

Twitter, a social media platform, has more than 320 million users worldwide and generates more than 500 million Tweets per day (27). The information produced through it is important because it has the potential to identify recent social trends and user behavior patterns (28). Therefore, we analyzed the relationship between potential keywords related to COVID-19 Omicron variant and vaccination and developed sentiment classification models for speech related to the Omicron variant pandemic by collecting Twitter data.

The results of topic modeling and CONCOR analysis showed that public opinions about vaccine effectiveness and breakthrough infection were formed on Twitter. Topic 2 of LDA topic modeling (vaccine) and cluster 3 of CONCOR analysis (vaccine effectiveness and the need for vaccine research) derived the same keywords such as effect, Pfizer, vaccine booster, prevention, antibody, and research. It is believed that it is because of people’s fear due to the occurrence of COVID-19 variants such as Delta variant and Omicron variant, as well as the decrease in vaccine efficacy according to the time elapsed after the primary series of the COVID-19 vaccines. Although Ouh et al. (29) reported that the effect that increased after the third COVID-19 vaccine dose began to decrease after 1 month of inoculation, it is difficult to conclude that vaccination was ineffective considering the statistics published by the Korea Disease Control and Prevention Agency (30), which showed that the group that completed the third COVID-19 vaccine dose had a 64–81% higher infection prevention effect, a 70–96% higher severe symptom prevention effect, and 95–99% higher mortality prevention effect.

In particular, breakthrough infection was derived from topic 4 of LAD topic modeling and cluster 2 of CONCOR analysis, where the breakthrough infection was defined as a confirmed COVID-19 case after 14 days from the day of completing vaccination (31). These results are thought to be because the currently prevalent Omicron variant spreads quickly due to its high contagious power and the immunity gained from vaccination or previous COVID-19 infection is less effective due to its immune evasion ability. The European Centre for Disease Prevention and Control (32) confirmed many mutations of the Omicron variant associated with increased transmission and immune evasion after natural infection and vaccination. Moreover, neutralizing antibody responses in sera sampled immediately after infection or vaccination were found to be less effective against the Omicron variant (33). It was also found that neutralizing antibody titers were reduced by up to 45-fold compared to the pandemic founder (33). It was confirmed that breakthrough infection cases were forming public opinions such as the uselessness of COVID-19 vaccines after the spread of the Omicron variant, which argued that vaccination was meaningless. However, from a different perspective, it may be a natural phenomenon that vaccinated people get COVID-19 more than unvaccinated people because more people are completing COVID-19 vaccination (34). In addition, Buchan et al. (35) evaluated the effect of vaccination on the Omicron variant and showed that the prevention effects of two doses against the Omicron variant decreased over time. However, they revealed that three doses of vaccine (booster shot) were effective in preventing the Omicron variant transmission. Therefore, it is expected that additional vaccination will be helpful to prevent the spread of COVID-19 variants. As such, conflicting opinions on vaccination on social media may create social anxiety and turmoil, and it also may exacerbate the public’s negative perception of vaccination. Although the results of this study inferred the reason for distrust in the efficacy of COVID-19 vaccination, it is still needed to analyze and share accurate information to alleviate negative factors such as public anxiety about vaccination.

We compared the predictive performance of the five sentiment classification models and confirmed that their accuracy was different according to the order of algorithm’s advancement (in the order of SVM, RNN, LSTM, BERT, and KoBERT). Especially, we confirmed that the difference in predictive performance between the SVM and RNN was the largest in this study because the SVM focused on binary classification and the SVM classification model was not linked to neural network computation. On the other hand, among deep learning models, the predictive accuracy of BERT was 5% higher than that of the RNN or LSTM. It was because the pre-trained natural language processing performed better in classifying actual speech. In other words, BERT showed faster and more accurate sentiment classification performance than the RNN and LSTM that were trained with a limited dataset in this study because BERT had more sophisticated embedding and induced more efficient computational processing than the RNN and LSTM, which were based on pre-training and computation. Moreover, in this study, KoBERT had 5% better predictive performance than BERT. It could be because although BERT and KoBERT are pre-training-based modes, the structure of the pre-trained text corpus and the corresponding tokenizer were optimized for learning Korean, and they performed better when the language is “Korean.” It is required to conduct more studies using BERT and KoBERT to understand the predictive performance of pre-trained-based models in actual speech including newly coined words.

Another finding of this study was that there was little difference in predictive performance between the RNN and LSTM. The LSTM is developed by improving gradient vanishing, gradient exploding, and long-term dependency problems of the RNN. Many previous studies (36–39) reported that it had superior predictive performance to the RNN. Nevertheless, it was found that the accuracy of the RNN and that of the LSTM were 62.24 and 62.89%, respectively. It could be explained in two ways. First, it was believed that there was no difference in accuracy between the RNN and LSTM because the long-term dependency problem rarely occurred. It was because speech data had short and simple sentence structures due to the character limit of “Twitter,” the data source of this study. Second, it contained more slang, newly coined words, and abbreviations (e.g., A.K.A.) due to the nature of speech in Twitter reflecting colloquial language compared to media and government announcements that showed a systematic structure and refined word choice of literacy language. As a result, the order of words did not contribute to semantic interpretation. Consequently, predictive performance was not different between the RNN and LSTM. Therefore, more studies are needed to examine deep learning-based NLP models targeting a corpus of various languages.

The importance of this study was to analyze people’s opinions about COVID-19 vaccination after the emergence of the COVID-19 Omicron variant in South Korea by conducting topic modeling and semantic network analysis using Twitter data. We also proved that pre-trained language models such as KoBERT greatly improved performance compared to the conventional RNN and LSTM, and presented basic data for the development of a future sentiment predictive model by comparing the performance of five sentiment classification models using unstructured data, which were meaningful results. The limitations of this study were as follows: First, Twitter limits the characters of the posting to 140. It has the advantage of maintaining the conciseness of the message, but at the same time, it may make it difficult to understand the message due to the use of abbreviations. Second, the results of this study could not be generalized and interpreted as general characteristics for the COVID-19 Omicron variant because this study used Tweets generated only for 3 months. It is necessary to continue monitoring because the Omicron variant is still ongoing. Also, in future studies, additional analysis including not only Twitter but also other social media is needed. Third, since the young generation mainly uses Twitter, the results of this study may not reflect the characteristics of the elderly who do not use Twitter. Fourth, the y-class of this study data (positive, negative, neutral) showed the characteristics of unbalanced data. Therefore, the unbalanced distribution of the y-class could play a key role in increasing the loss in processes such as weight selection in the learning process of the five NLP models. Particularly, there were more negative or neutral expressions than positive expressions due to the nature of an infectious disease or disaster—COVID-19. Fifth, due to the nature of the colloquial-oriented Twitter, the accuracy of predicting neutral or positive expressions is not high. It is believed that Tweets containing a negative characteristic could be classified confidently because they included strong negative meanings such as slang and direct wording (e.g., “not effective”). However, positive Tweets often contained neutral expressions, so it could make classification difficult. Future studies are needed to evaluate algorithms that can overcome the limitations associated with unbalanced data in embedding and weight selection calculation to improve the performance of NLP models that analyze colloquial speech such as Twitter.

## Conclusion

We performed topic modeling on Twitter users regarding the vaccination after the emergence of the COVID-19 Omicron variant by using Twitter data. The results confirmed that expectation, distrust, and anxiety regarding the efficacy of the vaccination coexisted: “Omicron,” “vaccine,” “vaccine inequality,” and “breakthrough infection.” Moreover, we developed sentiment classification models using five algorithms. The results confirmed that KoBERT showed the best performance (71%) in all predictive performance indicators (accuracy, precision, and F1 score). It is necessary to prepare measures to alleviate the negative factors of the public about vaccination in the long-term pandemic situation and help the public recognize the efficacy and safety of vaccination by using big data based on the results of this study.

## Data availability statement

The raw data supporting the conclusions of this article will be made available by the authors, without undue reservation.

## Ethics statement

The study was conducted according to the guidelines of the Declaration of Helsinki.

## Author contributions

GE, SY, and HB conceived the study and drafted the manuscript. GE collected the data. GE and SY performed the statistical analyses. HB critically reviewed and edited the manuscript. All authors contributed to data analysis, drafting, and revising the manuscript; gave final approval of the version to be published, have agreed on the journal to which the article has been submitted, and agreed to be accountable for all aspects of the work.

## References

[1] Yoo S Lim G. Analysis of news agenda using text mining and semantic network analysis: focused on COVID-19 emotions. *J Intell Inform Syst.* (2021) 27:47–64.

[2] World Health Organization. *WHO Coronavirus (COVID-19) Dashboard [Internet].* Geneva: WHO (2021).

[3] Ministry of Health and Welfare (South Korea). (2022). Available online at: http://ncov.mohw.go.kr/ (accessed April 22, 2022)

[4] Seong BL. Research and development of COVID-19 vaccine. *Orbis Sapientiae.* (2021) 30:117–27.

[5] Kim MR Kwon J Hong B. Current status of COVID-19 outbreak and vaccination in the Republic of Korea. *Int J Crisis Saf.* (2021) 6:34–44. 10.22471/crisis.2021.6.3.34

[6] Korea National Institute of Health. (2021). Available online at: https://nih.go.kr/gallery.es?mid=a20503010000&bid=0002&b_list=9&act=view&list_no=145484&nPage=1&vlist_no_npage=1&keyField=&keyWord=&orderby= (accessed April 22, 2022).

[7] World Health Organization. *Classification of Omicron (B.1.1.529): SARS-CoV-2 Variant of Concern.* (2021). Available online at: https://covid19.who.int/ (accessed April 22, 2022).

[8] Wolter N Jassat W Walaza S Welch R Moultrie H Groome M Early assessment of the clinical severity of the SARS-CoV-2 omicron variant in South Africa. *medRxiv* [Preprint]. (2021): 10.1101/2021.12.21.21268116 PMC8769664 35065011

[9] Lewnard JA Hong VX Patel MM Kahn R Lipsitch M Tartof SY. Clinical outcomes among patients affected with Omicron (B.1.1.529) SARS-CoV-2 variant in southern California. *medRxiv* [Preprint]. (2021): 10.1101/2022.01.11.22269045 PMC10208005 35675841

[10] Lee JA. *Incomplete Phrases Such as “Breakthrough Infection” in half of New Confirmed Cases Encourage “Vaccine Uselessness”.* (2021). Available online at: https://www.dongascience.com/news.php?idx=50998 (accessed April 25, 2022)

[11] Seo HR Song M. An analysis of the discourse topics of users who exhibit symptoms of depression on social media. *J Korean Soc Inform Manage.* (2019) 36:207–26. 10.1080/15295036.2019.1583349

[12] Prieto Santamaría L Tuñas JM Fernández Peces-Barba D Jaramillo A Cotarelo M Menasalvas E Influenza and measles-MMR: two case study of the trend and impact of vaccine-related twitter posts in Spanish during 2015-2018. *Hum Vaccin Immunother.* (2021) 4:1–15. 10.1080/21645515.2021.1877597 33662222 PMC9128558

[13] Ha SH Roh TH. Sentiment analysis for public opinion in the social network service. *J Converg Cult Technol.* (2020) 6:111–20.

[14] Lee SJ Kim HJ. Keyword extraction from news corpus using modified TF-IDF. *J Soc Bus Stud.* (2009) 14:59–73.

[15] Blei DM Ng AY Jordan MI. Latent dirichlet allocation. *J Mach Learn Res.* (2003) 3:993–1022.

[16] Kim YH. *Social Network Anaysis.* Seoul: Pakyongsa Press (2016).

[17] Borgatti SP Everett MG Freeman LC. *Ucinet 6 for Windows: Software for Social Network Analysis.* Harvard, MA: Analytic Technologies (2002).

[18] Son DW. *Social Network Anaysis.* Seoul: Kyungmoonsa (2002).

[19] Hyunh PH Nguyen VH Do TN. Enhancing gene expression classification of support vector machines with generative adversarial networks. *J Inf Commun Converg Eng.* (2019) 17:14–20.

[20] On BW Park SM Na CW. *KNU Sentiment Lexicon.* (2018). Available online at: https://github.com/park1200656/KnuSentiLex (accessed April 22, 2022).

[21] Mikolov T Karafiát M Burget L Cernocký J Khudanpur S. Recurrent neural network based language model. *Interspeech.* (2010) 2:1045–8. 10.21437/Interspeech.2010-343

[22] Wei D Wang B Lin G Liu D Dong Z Liu H Research on unstructured text data mining and fault classification based on RNN-LSTM with malfunction inspection report. *Energies.* (2017) 10:406. 10.3390/en10030406

[23] Peters ME Neumann M lyyer M Gardner M Clark C Lee K Deep contextualized word representations. *arXiv* [Preprint]. (2018): 10.18653/v1/N18-1202

[24] Radford A Narasimhan K Salimans T Suts-kever I. *Improving. Language Understanding by Generative Pre-Training*. (2018). Available online at: http://cdn.openai.com/research-covers/language-unsupervised/language_understanding_paper.pdf (accessed April 22, 2022).

[25] Devlin J Chang MW Lee K Toutanova K. BERT: pre-training of deep bidirectional transformers for language understanding. *arXiv* [Preprint]. (2018):

[26] Korean Bert pre-trained cased (KoBERT). (2022). Available online at: https://github.com/SKTBrain/KoBERT (accessed April 22, 2022).

[27] Aslam S. *Twitter by the Numbers: Stats, Demographics & Fun Facts.* (2022). Available online at: https://www.omnicoreagency.com/twitter-statistics/#:~:text=Twitter%20Demographics&text=There%20are%20262%20million%20International,users%20have%20higher%20college%20degrees (Accessed April 22, 2022)

[28] Lee CH Hur J Oh HJ Kim HJ Ryu PM Kim HK. Technology trends of issue detection and predictive analysis on social big data. *Electr Telecommun Trends.* (2013) 28:62–71.

[29] Ouh IO In HJ Lim HJ Park HJ Kim BC Kim SS Latest trend of the COVID-19 booster vaccination. *Public Health Wkly Rep.* (2022) 15:556–64.

[30] Korea Disease Control and Prevention Agency. (2022). Available online at: https://ncv.kdca.go.kr/menu.es?mid=a10116020000 (Accessed April 22, 2022).

[31] Ahn SH Lee SH. Updates on coronavirus disease 19 vaccine and its clinical application. *Korean J Fam Pract.* (2021) 11:236–46. 10.21215/kjfp.2021.11.4.236

[32] European Centre for Disease Prevention and Control. *Implications of the Further Emergence and Spread of the SARS-CoV-2 B.1.1.529 Variant of Concern (Omicron) for the EU/EEA.* (2021). Available online at: https://www.ecdc.europa.eu/sites/default/files/documents/threat-assessment-covid-19-emergence-sars-cov-2-variant-omicron-december-2021.pdf (Accessed June 10, 2022)

[33] Sheward DJ Kim C Ehling RA Pankow A Dopico XC Martin D Variable loss of antibody potency against SARS-CoV-2 B.1.1.529 (Omicron). *bioRxiv* [Preprint]. (2021): 10.1101/2021.12.19.473354

[34] Centers for Disease Control and Prevention. *Possibility of Covid-19 Infection After Vaccination: Breakthrough Infection.* (2021). Available online at: https://korean.cdc.gov/coronavirus/2019ncov/vaccines/effectiveness/why-measure-effectiveness/breakthrough-cases.html#anchor_1636141951606 (accessed April 22, 2022).

[35] Buchan SA Chung H Brown KA Austin PC Fell DB Gubbay JB Effectiveness of COVID-19 vaccines against omicron or delta symptomatic infection and severe outcomes. *medRxiv* [Preprint]. (2022): 10.1101/2021.12.30.21268565 PMC9500552 36136332

[36] Kim SW. Forecasting COVID-19 pandemic stock prices using portal search intensity and deep learning. *J Digit Contents Soc.* (2022) 23:343–50. 10.9728/dcs.2022.23.2.343

[37] Kim JW Jo HI Lee BGA. Comparison study on performance of malicious comment classification models applied with artificial neural network. *J Digit Contents Soc.* (2019) 20:1429–37. 10.9728/dcs.2019.20.7.1429

[38] Shin DH Choi GH Kim CB. Deep learning model for prediction rate improvement of stock price using RNN and LSTM. *J Korean Inst Inform Technol.* (2017) 15:9–16. 10.14801/jkiit.2017.15.10.9

[39] Cho YB Kim KJ Ku JA Woo SH. Change of malicious code API call pattern extraction using RNN and LSTM. *Int Conf Inf Commun Eng.* (2019) 11: 277–80.

